# Using MMI Comments for Medical School Admissions Decision-Making

**DOI:** 10.15694/mep.2018.0000098.1

**Published:** 2018-05-11

**Authors:** Rubia Khalak, Linnie Newman, Julia Saltanovich, Laurie Thibodeau, Sarah E. McCallum

**Affiliations:** 1 Albany Medical Center; 2 Albany Medical College

**Keywords:** Multiple mini interview, Medical school admissions

## Abstract

This article was migrated. The article was marked as recommended.

Introduction

Multiple mini-interview (MMI) comments can help to reveal an underlying personality or behavioral flaw that the numerical scores are not designed to illustrate. The importance of the comments provided by interviewers has not been documented in the literature. This study sought to examine whether MMI comments influenced admission committee decision-making.

Methods

Five thousand de-identified interview comments from a subset of 625 medical school applicants were reviewed by study raters who followed a rubric that outlined scoring assignments based upon the number of positive and negative statements made by MMI interviewers. The presence of extremely negative MMI comments was also evaluated.

Results

MMI score strongly correlated with MMI comment score assigned by the raters and contributed to overall committee score and decision for acceptance or rejection to medical school. The presence of one negative outlier comment score alone did not impact committee score or acceptance; however, when extremely negative attributes were identified by study raters within an outlier comment, this was associated with a reduction of final committee score and higher incidence of rejection.

Conclusions

A strong correlation existed between the MMI comments and overall MMI score. Both MMI comments and MMI scores predict acceptance to medical school. The additional value of the MMI comments lies in the extremely negative outlier comments.

## Introduction

The Michael DeGroote School of Medicine at McMaster University developed the Multiple Mini-Interview (MMI) to address two widely recognized problems: increased criticism of the traditional interview format for not being able to accurately predict performance in medical school, and that patient complaints involved physician’s performance related to interpersonal skills, professionalism and ethical/moral judgment ( Eva et al. 2004a). These non-quantitative skills set may be more difficult to evaluate in a one-on-one interaction regardless of the length of the interview.

Researchers at McMaster University have reported that the use of the MMI has increased the reliability of the interview in assessing a candidate’s suitability for the practice of medicine ( Eva et al. 2004b). Eva et al concluded that the MMI was a reliable tool to evaluate the personal qualities of interview candidates by providing “context specificity” with the multiple interviewer method. The authors also showed that having a heterogeneous interviewer pool from health science faculty members to community members provided an increase in diversity of the group of accepted applicants. The Medical College Admissions Test (MCAT) score and undergraduate grade point average (GPA) alone do not accurately reflect how well a candidate will be able to converse and interact with patients during medical school clerkships or as a future physician. The MMI can be used as a preferred interview format to tease out these all-important metrics by differentiating those applicants who may perform well on a subjective one-on-one medical school interview but falter with at least one of the multiple structured, objective mini-interviews ( Saguil et al. 2015; Lemay et al. 2007; Yeates et al. 2015; Donnon et al. 2008).

The MMI process involves both numeric scores for assessment as well as written comments by the interviewer. Although the literature is replete with studies evaluating the overall MMI process ( Pau et al. 2013; Eva et al. 2012; Husbands et al. 2013; Oliver et al. 2014), there is yet nothing reported on the use of MMI comments. The impact of comments as an evaluative tool has been investigated in other medical education settings. Ginsburg and colleagues described efforts to score evaluative comments on residency reports (2012; 2016; 2017), and found that written comments often supply additional, valuable information that is not replicated by a quantitative measure such as a Likert score (2011; Durning et al. 2010). This study had a similar methodology where descriptive written comments were converted to a numerical score and compared to both the overall MMI score as well as to the final admissions committee ranking score which determines acceptance or denial. In addition, this study evaluated the contribution of outlier MMI scores and particularly negative comments on the overall admissions committee ranking score and decision of whether to admit or reject.

The objective of this study was to investigate whether MMI interviewer comments had an impact on admissions committee decision-making. A single numeric score without contextual framework often makes it difficult for the admissions committee members to assess the parameters that had been used to assign the MMI score. The MMI score is entered by the interviewer using a 1 to 5 Likert scale, placing many students in the “middle” or “3” category. MMI comments may be used to differentiate applicants in this group. Of additional importance is a score of a “1” or “2” indicating a poor or weak candidate. Specific MMI comments explaining the assigned score can be used by the admissions committee to decide selection or rejection. We also hypothesized that MMI comments rather than only the numeric score would identify personality traits or behavioral concerns that the MMI was designed to capture. The MMI comments could guide decision-making for medical school admissions and an extremely negative MMI comment would itself have an impact on decision-making.

## Methods

**Figure 1.**
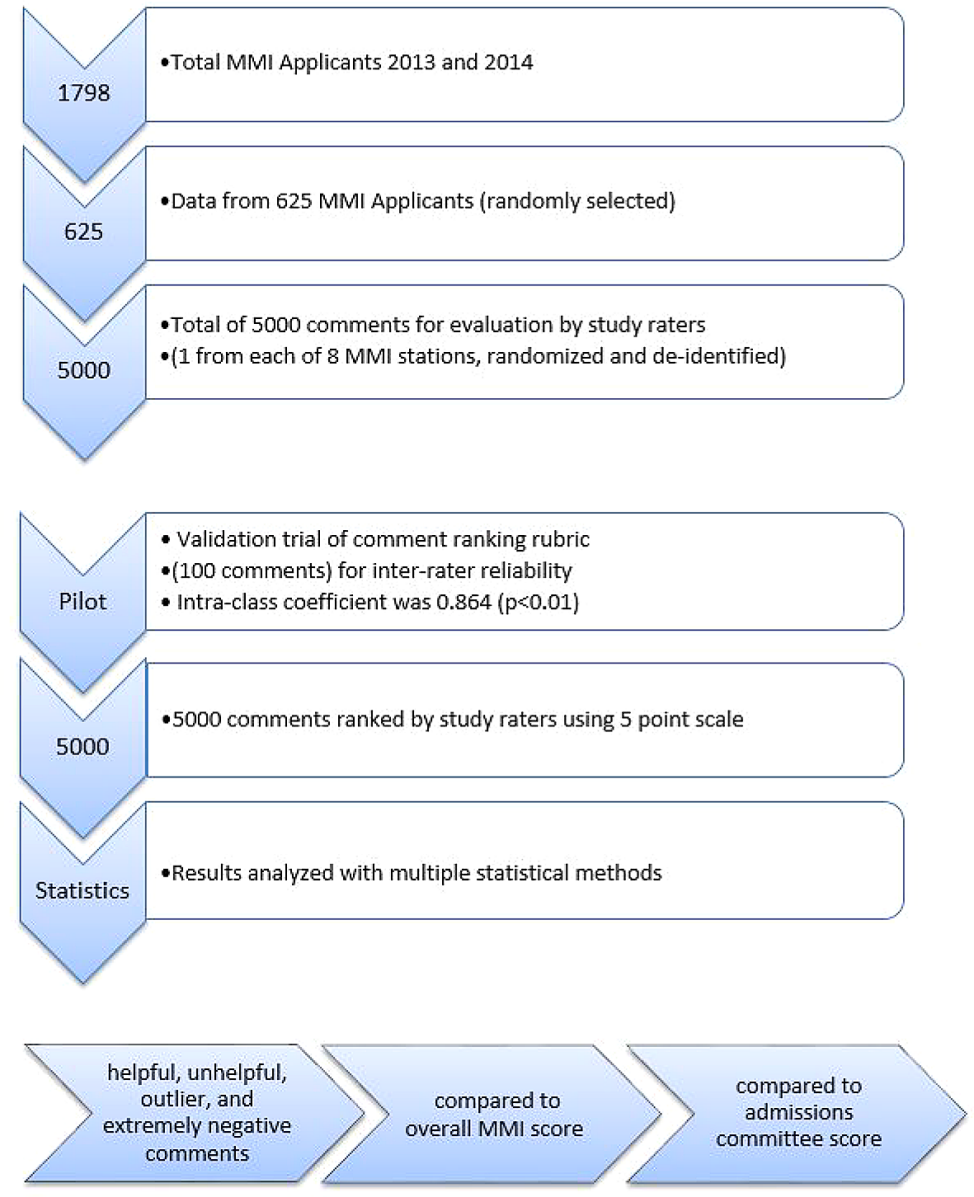
Flow Diagram of Observational MMI Study

This retrospective observational study was approved by the Institutional Review Board at Albany Medical College (AMC) under reference number #4592. A total of 1798 applicants were interviewed in the 2013 and 2014 application cycles, Figure 1. The de-identified interview profiles of 625 students were randomly selected for review, resulting in 5000 individual MMI interviewer comments to be evaluated. This represented eight interviewer comments per applicant, one comment for each of eight individual MMI scenarios. Each applicant was assigned a random identification number. We selected about 300 applicants from each application year. The number of students was chosen to provide a large yet manageable number of comments for study raters to evaluate. Excluded from analyses were medical school applicants who were not interviewed through the MMI process, such as students applying to one of the combined degree programs. De-identified data included demographics data such as gender, age, race, state of residence, and if applicant was a re-applicant. Evaluative measures collected included overall GPA, science GPA, total MCAT score, MMI interview score, study comment score of each MMI station (see below) and overall admissions committee ranking score.

### MMI process

Each applicant participated in eight MMI stations, each involving a specific scenario with a single interviewer, and each lasting ten minutes. Applicants had two minutes to read the scenario and a maximum of eight minutes to discuss with the interviewer. Scenarios were comprised of six categories: personal attributes, physician attributes, public policy, social justice, knowledge and understanding of complementary/alternative medicine, and communication/empathy. The communication/empathy station was assessed by observing the candidate interact with a standardized patient playing a specific role in the scenario.

MMI interviewers consisted of faculty, college administrators, medical students, and other AMC community members blinded to any information about the applicant. Interviewers were given standardized preparation on assessment parameters and trained on the use of scoring rubrics. MMI interviewers were instructed to provide comments that described both content about the scenario and applicant attributes. Specifically, interviewers were asked to focus their comments on an applicant’s ability to respond to the case prompts, also taking into consideration the body language, eye contact, and attitude of the applicant. Interviewers were also instructed to avoid any reference to race, ethnicity, or personal descriptors in the comments. Finally, interviewers were instructed to use the full range of the Likert scale.

At the completion of the MMI sessions, MMI scores including comments were sent to the admissions committee. Additional data were also provided to the admissions committee members, including the mean scores given by an interviewer for the eight applicants seen that day, although the identity of the MMI interviewers was kept hidden.

### Study rating of MMI comments

**Figure 2.**
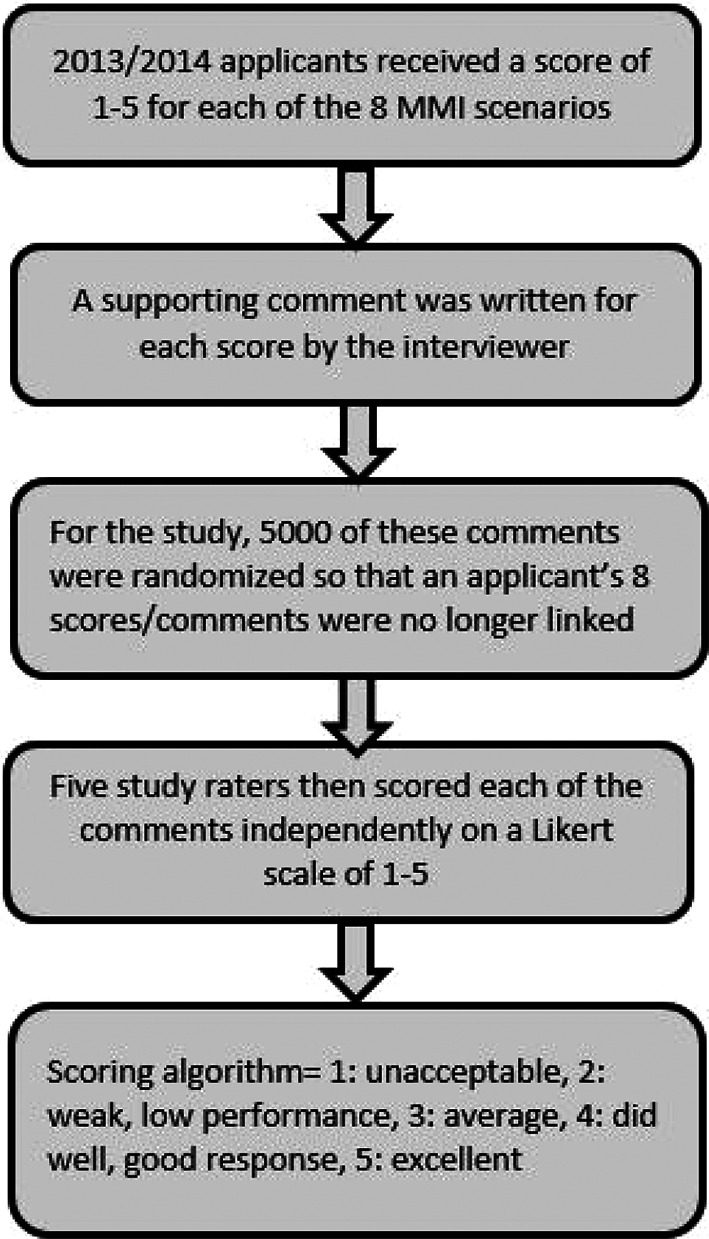
Process of Scoring MMI Comments by Study Raters

A randomization of all comments within each application cycle was done to prevent bias of comment scoring when comments were previously grouped by applicant. With MMI interviewer scores and all applicant information hidden, five study raters evaluated each of 5000 comments rating the comment itself on a scale of 1 to 5 based on the quality of the applicant’s answer to the scenario and the applicant’s demeanor in the scenario, as shown in Figure 2. To verify the validity of the rating rubric, interrater reliability among the five comment raters was measured after evaluating 100 random comments and the intra-class coefficient was determined to be 0.864 (p<0.01).

After unscrambling and re-grouping the comments by applicant, each de-identified applicant’s set of comments was evaluated for the presence of an outlier comment. Outlier comments were defined as those receiving a study rater score that was more than 1.5 standard deviations from the mean score of all eight comments given to that applicant. Outlier comments were also assessed for impact on the final committee decision for acceptance or rejection. Finally, the five study raters evaluated de-identified and randomized outlier comments for the presence of extremely negative qualitative comments described by the MMI interviewer, such as lack of empathy, paternalism, rigidity, aggression, or extreme awkwardness during a scenario.

### Statistical Analysis

Descriptive statistics were used to compare demographic characteristics, including age, gender, and re-applicant status. Statistical analyses included Chi-square tests of categorical variables and Pearson correlations to examine the relationship between continuous variables. Both paired and independent t-tests were performed to determine if either MMI score differed from study rater comment score for an applicant, or, to compare MMI scores and study rater comment scores among demographic and predictor variables such as gender. Multiple linear regression was used to determine the predictive validity of MMI comments on final ranking committee score. The level of significance was established at p < 0.05. All statistical analyses were performed using SPSS software (Version 23, IBM Corporation, Armonk, NY).

## Results

As summarized in Table 1 of demographic information, there were no differences in age or gender in the applicants when comparing the two study years. Table 2 shows examples of interviewer comments that study raters felt were particularly helpful in assigning the rater scores. Out of 5,000 comments, approximately 10% were identified as unhelpful, as they merely stated the applicant’s answer to the scenario but failed to adequately characterize either the specific content of the applicant’s response or describe the applicant demeanor during the interview.

**Table 1.** Descriptive statistics for demographic data and variables used in regression model (n = 625)

Female, n (%)	239 (38.2%)
Reapplicant, n (%)	363 (58.1%)
Accepted, n (%)	176 (28.2%)
Out of State, n (%)	479 (76.6%)
Age, mean ± SD (range)	24.56 ± 2.19 (20.0-38.0)
MCAT, mean ± SD (range)	32.03 ± 2.94 (20-39)
GPA, mean ± SD (range)	3.51 ± 0.26 (2.54-4.0)
Science GPA, mean ± SD (range)	3.42 ± 0.32 (2.27-4.0)
MMI score, mean ± SD (range)	3.52 ± 0.58 (1.50-4.88)
MMI comment score, mean ± SD (range)	3.23 ± 0.46 (1.40-4.43
Final ranking committee score, mean ± SD (range)	410.02 ± 37.10 (100.0-475.0)

**Table 2.** Helpful and unhelpful MMI comments with interviewer and study raters’ scores

MMI Interviewer score	Helpful Comments
**5**	An excellent interview; professional; articulate; passionate about his choice of organization; very insightful; a great discussion and easy conversationalist. A strong applicant.
**4**	He seemed thoughtful and concerned about what is best for the patient as well as the family. He was articulate and able to give well supported answers.
**3**	The applicant was able to show empathy in an appropriate manner and was able to identify the unethical portion of the scenario.
**2**	Didn’t take a strong position on either side..... waffled in her responses.... and in the closing seconds tried to bring the discussion around to how this scenario relates to the doctor/patient relationship. I’m still struggling to follow that particular bit of thinking.
**1**	This applicant mumbled and rambled through the scenario of physician shortage. He was not able to articulate his thoughts and stop at appropriate times. His eye contact was also bad. I would not feel comfortable with this student in our class.
	**Unhelpful Comments**
**4**	Topic on Mental Illness
**2**	Thought that the nursing home would be the best disposition for the woman with brain damage, and was prompted to consider other approaches and implications of that decision

Mean MMI comment score (± SD) refers to the independent study rater score whereas the MMI score refers to the numerical Likert scale score assigned by the interviewer. Using a paired t-test approach, the mean MMI comment scores derived from study raters’ assessment of MMI comments was lower than the mean MMI scores given by the interviewer, 3.23 vs 3.52; p < 0.05. Nevertheless, there was a strong correlation between rater MMI comment score and MMI interviewer score (r = 0.91; p < 0.01). MMI scores for female applicants were higher than those for male applicants, 3.62 (± 0.04) vs 3.47 (± 0.03); p < 0.01. Interestingly, the study rater MMI comment scores of female applicants were also higher than those of males (3.33 (± 0.03) vs 3.18 (± 0.02); p < 0.01) despite study raters not having explicit information about applicant gender during the scoring process.

We found that accepted students had both a significantly higher mean MMI score and higher MMI comment score. The mean MMI score among accepted students was 3.75 (± 0.54), while the mean MMI score among the students who were not accepted was 3.43 (± 0.58), (t (623) = 6.25; p < 0.01). Similarly, study raters’ assessments of the MMI comments were higher in those students who were accepted, 3.4 (± 0.43) versus those who were not accepted, 3.17 (± 0.45); t (623) = 5.89; p < 0.01. There were strong correlations between admissions committee score and both the MMI score and MMI comment score (r = 0.51; p < 0.01 and 0.50; p<0.01, respectively).

### MMI comments predict admission committee scores

Multiple regression analyses were performed to determine whether MMI comments were a better predictor of final admissions committee score than commonly used academic evaluative variables such as GPA and MCAT. Pearson correlations were calculated for multiple predictor variables and the following variables were found to be significantly correlated with committee score (p < 0.05): MMI score, MMI comment score, MCAT, GPA, science GPA, and age. After examining tolerance and variance inflation factors to adjust for multicollinearity, and removing highly correlated predictor variables from the model, the following variables were analyzed in the regression model: MMI comment score, total MCAT, and overall GPA. Demographic variables were controlled for, including age, gender and whether the student was a re-applicant. The fit of the regression model with and without MMI comment score was evaluated, Table 2. Control variables (GPA, MCAT, gender, age, re-applicant status) contributed to an R ^2^ = 0.07, meaning that these variables only predicted 7% of the variance in committee score. The addition of MMI comment score increased the R ^2^ to 0.30, meaning 30% of the variance in committee score was predicted when MMI comment score was included in the model. As MMI score and MMI comment score were highly correlated, they could not be analyzed in the same model; however nearly identical results were obtained using MMI score in place of MMI comment score (R ^2^ = 0.32).

### Influence of MMI outlier scores and comments

Outlier MMI scores were analyzed to determine the extent to which they influenced the acceptance of that applicant to medical school. Of the 625 applicants studied, 279 applicants (45%) received an outlier MMI score, which was defined as one of eight MMI scores per applicant falling 1.5 SD beyond the mean MMI score for that applicant. The presence of an outlier MMI score itself did not impact applicant acceptance to medical school (X² = 0.13; p > 0.05), nor did it effect the mean admissions committee score. For these applicants, one weak performance at an MMI station was balanced by the other stronger MMI station performances and did not significantly impact the final admissions committee score.

The 279 applicants with outlier MMI scores had their MMI comments re-analyzed by the study raters to assess if there was a subset of these applicants who had particularly negative comments linked to their outlier MMI score that possibly did affect the admissions committee score. This review found that 82 applicants (13% of the 625 applicants) were identified by at least three of the five study raters as receiving an extremely negative comment. Examples of comments displaying a strongly negative attribute are *“No empathy or acknowledgment”, “Told his own story, not supportive.”, “He was judgmental both verbally and non-verbally”.* When strongly negative attributes were present within an outlier comment, applicant committee score was indeed significantly lower, 400.93 ± (39.64) compared with committee scores for applicants with outlier comments not containing this degree of extremely negative content, 415.56 (± 31.69); p < 0.01). Examples of interviewer comments that were outliers but not determined to be extremely negative are “Offered minimal explanation for his position.”, “Did not convey confidence in her decision”. Chi-square analysis confirmed that the presence of an extremely negative outlier comment led to more applicant rejections, compared to outlier comments not containing negative language (X ^2^ = 10.77; p < 0.01).

## Discussion

Interviewer comments provided during the MMI are a valuable part of the medical school selection process. Our study found a high correlation between MMI interviewer comments and MMI numerical scores, suggesting that MMI comments reliably reflect MMI numerical scores. This finding of the study was not unexpected as interviewers receive orientation on MMI interviewing, evaluation and instruction on comment completion. One notable exception may be when unhelpful comments are provided, and include only content about the scenario or attributes of the applicant, but not both. Moreover, in multiple regression models, both MMI comment scores and MMI scores predicted final admissions committee score, which determined acceptance to medical school. Higher MMI scores were noted for female applicants when compared to male applicants, resulting in a higher rate of acceptance, consistent with what has been previously reported ( Ross et al. 2017). The effect of gender persisted even when de-identified interviewer comments were evaluated by independent study raters blinded to the applicant’s gender. A potential explanation for this result is unclear and warrants further study.

On occasion, an applicant would receive a single score that was a statistical outlier from the mean score of that applicant, such as a “2” amid mostly 4’s and 5’s. The presence of an outlier comment score alone did not impact the overall admissions committee score or acceptance to medical school. This is reasonable, as one lower performing interview station amongst strong performing ones should have less of an impact on the overall score. However, when the outlier score was accompanied by an extremely negative attribute, the resulting final committee score was significantly lower and incidents of rejection higher than when the comment was not identified by study raters as extremely negative. This finding emphasizes the importance of both written comments, particularly strongly negative comments in assessing the MMI. Although the presence of extremely positive comments was not analyzed in the same rigorous fashion, such MMI comments were accompanied by high scores.

Qualitative comments have been examined extensively in the context of medical resident and clerkship evaluations ( Ginsburg et al. 2017; Cook et al. 2016). However, narratives used in the context of the medical school selection process have not been systematically evaluated. We observed a strong correlation between MMI interview scores and comments. This shows that comments and numerical scores may be used to a similar extent in the selection process. Interestingly, in a linguistic analysis of resident reports, Ginsburg and colleagues found that faculty evaluations often hedged in their statements to “save face” for themselves and the residents, noting that training faculty to provide more helpful or “balanced” comments of strengths and weaknesses did not offer any improvement in the reports as balanced comments were thought to signal a weak resident ( Ginsburg et al. 2016). We found that more negatively worded comments did impact admissions committee decision-making. While a linguistic analysis was not performed in our study, the observation that strongly negatively worded statements provide more meaning to admission committees than did neutral comments may be partially explained by the interviewer anonymity inherent in the MMI process. This anonymity may allow for accurate evaluations by the interviewer without the need to “save face”. Indeed, the present findings are complementary to work by Durning and colleagues, who demonstrated that program director evaluations of medical students provided additional value over numerical scores, particularly when evaluative comments were negative in nature ( During et al. 2010).

A limitation of our study was its retrospective design. A prospective study would have allowed access and collection of data not usually retained during an MMI such as the particular scenarios used on a specific interview date and comparison of scores by specific MMI interviewer in a de-identified manner. In addition, less than 40% of applicants reviewed for this study were female. In the applicant pools for the years of the study, females comprised 48% of all applicants. One strength of this study was the large database and sheer number of comments available for review. A second strength was the strong inter-rater reliability amongst the five independent study raters when scoring MMI comments. The study raters were all familiar with the MMI process but had varying degrees of involvement in medical school admissions. Despite this, study raters independently developed similar reviews and critiques.

**Table 3.** Multiple Linear Regression With and Without MMI Comment Score

Model	Unstandardized Coefficients		Standardized CoefficientBeta	t	Sig	95% Confidence Interval for B	
	B	SE				Lower	Upper
1.(Constant)	362.12	33.97	---	10.66	.000	295.42	428.82
UG GPA	15.59 ^*^	6.01	0.11	2.60	.010	3.80	27.39
MCAT	1.20 ^*^	0.50	0.10	2.39	.017	0.21	2.18
REAPPLICANT	-4.04	3.10	-0.05	-1.30	.193	-10.12	2.05
AGE	-1.90 ^**^	0.71	-0.11	-2.67	.008	-3.30	-0.50
GENDER	10.41 ^**^	3.03	0.14	3.44	.001	4.46	16.36
2. (Constant)	205.46	31.32	-------	6.56	.000	143.96	266.96
UG GPA	20.64 ^**^	5.21	0.14	3.96	.000	10.42	30.86
MCAT	1.176 ^**^	0.43	0.93	2.72	.007	0.327	2.03
REAPPLICANT	-4.612	2.68	-0.61	-1.72	.086	-9.88	0.65
AGE	-1.42 ^*^	0.62	-0.84	-2.30	.022	-2.64	-0.21
GENDER	4.65	2.65	0.06	1.75	.080	-0.56	9.85
MMI COMMENT ^1^	40.30 ^**^	2.79	0.49	14.44	.000	34.82	45.78

^1^ MMI score was not entered into the regression model as too highly correlated with MMI comment score. In a separate regression model, MMI score contributed to the variance in committee score. R ^2^ in the absence of MMI score was 0.067 and increased to 0.315 when MMI score was added to the model. *p < 0.05; **p < 0.01

## Take Home Messages

### CONCLUSION

The MMI is designed to help identify qualities in medical school applicants such as empathy and social awareness that may otherwise be unrecognized by traditional assessments. This study shows that MMI comments, when combined with numerical scores, provide additional valuable information in medical admissions decision-making, especially when interviewers are instructed to write comments that describe both attributes and information about how the applicant responded to the scenario. In addition, when an extremely negative comment is present, it is the actual interviewer comments themselves, not the numerical score that impact the admissions committee ranking decision. It is therefore crucial that medical schools that using the MMI both train and remind their interviewers to write specific comments that align with the numeric score. Comments should include both information about the candidates’ ability to discuss a scenario and their communication and interpersonal skills.

### Practice Points:

• This study shows that MMI score strongly correlated with MMI comment score assigned by the raters and contributed to overall committee score and decision for acceptance or rejection to medical school.
• In addition, when an extremely negative comment is present, it is the actual interviewer comments themselves, not the numerical score that impact the admissions committee ranking decision.
• Therefore, it is crucial that medical schools using the MMI train and remind their interviewers to write specific comments with both information about the candidates’ ability to discuss a scenario and their communication and interpersonal skills.

## Notes On Contributors

RK contributed to concept and design of the study, interpretation of data, and the writing of paper. RK is a member of the Executive Admissions Committee.

LN contributed to the design of the study. LN is an associate dean of the medical school and a member of the Executive Admissions Committee.

JS contributed to the drafting of paper. JS is assistant director of admissions and a member of the Executive Admissions Committee.

LT contributed to the interpretation of the data and drafting of paper. LT is director of undergraduate education.

SM contributed to the design of the study, analysis and interpretation of the data and writing of paper. SM is a member of the Executive Admissions Committee.

All authors contributed to the rating of MMI comments and critical revision of paper. All authors approved the final manuscript for publication and agree to be accountable for all aspects of the work.
