# Supplementary material for: Using MMI Comments for Medical School Admissions Decision-Making
Source: MedEdPublish (2016). 2018 May 11;7:98. [Version 1] doi: 10.15694/mep.2018.0000098.1 (PMC10711995; doi:10.15694/mep.2018.0000098.1)
Supplement: Peer review history

# Reviewer report: Reviewer response for version 1

**DOI:** 10.21956/mep.19565.r29020

**Published:** 2019-03-14

**Authors:** Richard Hays

**Affiliations:** 1 James Cook University

This review has been migrated. The reviewer awarded 4 stars out of 5 The authors are to be commended for this study. Although the findings might be regarded as ‘expected’, it is nice to see confirmation through a formal process. It is reassuring that comments reflect the scores, although raises questions about how the comments could be used in formal processes. Some programs state that comments may be used in borderline decisions about applicants, but this study suggests that doing that might be redundant and perhaps unfair ‘double jeopardy’. All programs should consider conducting this kind of analysis from time to time as a QA activity.

Reviewer Expertise:

NA

No decision status is available

# Reviewer report: Reviewer response for version 1

**DOI:** 10.21956/mep.19565.r29019

**Published:** 2019-03-02

**Authors:** Ken Masters

**Affiliations:** 1 Sultan Qaboos University

This review has been migrated. The reviewer awarded 5 stars out of 5 An interesting paper on a mostly-ignored topic, probably because only the scores “count”, and the comments are frequently ignored. The authors, however, have highlighted the need to assess the comments as well as the scores, even if to understand the central tendency in the reviewers’ ratings.The relatively large sample of 625 students provides a robust base for the statistical analyses. The detailed analysis of the data is welcomed, and the authors have taken great care in ensuring the validity of their conclusions.The strong correspondence between comments and score could possibly have been predicted, but also serves to give the school confidence in their scoring system, ensuring that scores are not arbitrarily assigned. The analysis of outliers in light of the comments is also of value, as it indicates a weakness that should be addressed, but would not be enough to warrant exclusion.Overall, a well-written an insightful paper.

Reviewer Expertise:

NA

No decision status is available

# Reviewer report: Reviewer response for version 1

**DOI:** 10.21956/mep.19565.r29018

**Published:** 2018-05-18

**Authors:** Sateesh Babu Arja

**Affiliations:** 1 Avalon University School of Medicine

This review has been migrated. The reviewer awarded 5 stars out of 5 It is a very well written and evidence-based research article. It is very thoughtful and provided the insight on the mini-multiple interview process for selection of medical students. Nowadays there is a lot of discussions regarding a need to change the interview process. Many Medical Schools are drifting from traditional interviews to mini-multiple interviews and situational judgment tests which have better validity and reliability compared to the traditional interviews. This paper adds the value to the existing literature on mini-multiple interviews. This paper also suggests the importance of narrative comments rather than just the ratings by the interviewers. Ratings based on the narrative comments might be a better process of selection of medical students as the medical profession expects high levels of communication and professionalism from the medical graduates. It is also good and this paper shows that ratings given by the interviewers are correlating with ratings that were given based on narrative comments.I would also like to add my comment that most of the personal attributes that we are looking in the interview process like Mini-multiple interviews are unstable and some of the behaviors are learned by medical students during the medical program and it is also the responsibility of the medical programs to teach those behaviors. The other comment I would like to add is that in this it was shown that MMI scores are high for females and so the acceptance rate. I would like to see if the females also have high GPA or MCAT scores compared to males in this study?This is a very interesting article for me as I am involved in the admissions process as an academic administrator. This article is very interesting for interviewers, the admissions committee members, and the academic administrators involved in the admissions and selection of medical students.

Reviewer Expertise:

NA

No decision status is available
